# Stromules: An Incident or Formalized Pathway for Molecules Transfer Between Organelles?

**DOI:** 10.3390/ijms262110680

**Published:** 2025-11-02

**Authors:** Joanna Grzyb

**Affiliations:** Department of Biophysics, University of Wrocław, Joliot-Curie 14a, 50-383 Wrocław, Poland; joanna.grzyb@uwr.edu.pl

**Keywords:** stromules, plastids, envelope, retrograde signaling, immune response, endoplasmic reticulum

## Abstract

The stromules are tubular extensions of chloroplasts, or broader plastids, formed by the organelle envelope and filled with the stroma, the internal content of organelle. The formation of stromules is related to the cytoskeleton. Stromules occur in photosynthetic tissues under illumination and are therefore proposed to be important for retrograde signaling, which is essential for adaptation to stress factors. Stromules are also observed after pathogen attack. Some groups propose that stromules are a resemblance to endoplasmic reticulum dynamics, without having actual significance in molecules transport within a cell. However, there is no consensus among researchers regarding the actual function and significance of stromules, which can be the result of different models used to study stromules, and the necessity of using fluorescent labels, with all advantages and limitations of fluorescence-based methodology. Here I briefly discuss current knowledge on the subject from a perspective of stromule origin—the chloroplast envelope, and the potential advantages of having a conduit within organelles instead of relying on diffusion through cytosol.

## 1. Introduction

The stromules are among of the most intriguing structures that can be occasionally found in a plant cell. In the literature, stromules are described as tubular extensions of chloroplasts or, more broadly, plastids, and there is no conssensus among researchers regarding their actual function and significance. The diameter of stromules is within a range of 0.35–0.85 μm, while the length can reach be hundreds of micrometers [1,2]. The Reader may find several reviews, focused on stromules over the last two decades [3,4,5,6,7], with the recent one by Jung et al. [8]. From this perspective, one may trace how our understanding of stromules function changed, with no clear conclusion reached. The reason for that is still relatively low (although growing), compared to other subjects, number of studies of devoted stromules. It might be noted that shortly after discovery, stromules were proposed to serve as communication pathway between chloroplasts and other organelles, while recently, the research focuses on stromules in plant immune response, with the thesis that they primarily function in those situations [9]. Over the years, there were hypotheses including stromules’ involvement in nutrient (particularly sugar) sensing [10] and exchange of components of chloroplasts/other organelles [11]. Some researchers also postulate that stromules are a negligible result of the interaction between dynamic endoplasmic reticulum (ER) and the chloroplast envelope [12,13,14].

Studies on stromules have been made possible through the use of fluorescent proteins, as native stromules are colorless. For a very good overview of this methodology—and its derivative application—in the study of stromules and other related structures, the Reader may refer to a mini-review by Baillie et al. [15].

**Figure 1:**
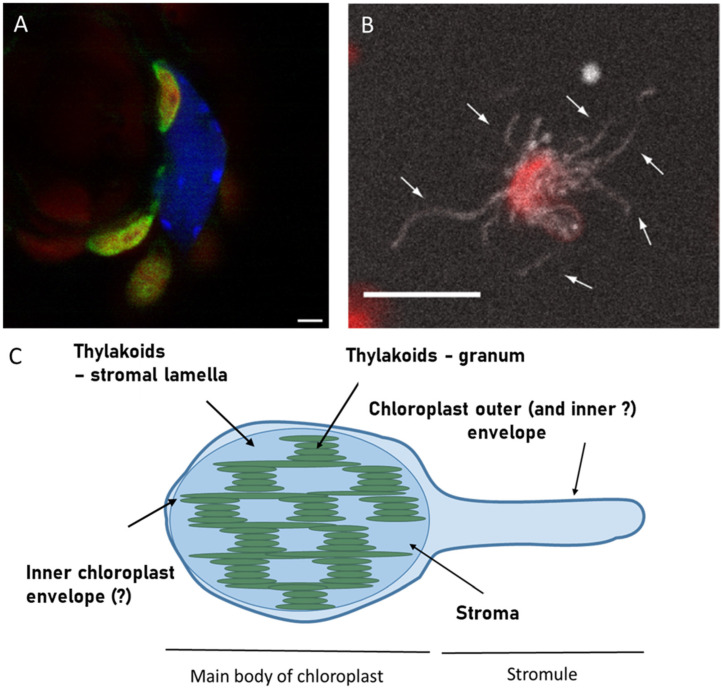
Example of stromules CLSM observation in Arabidopsis thaliana seedlings using GFP labelled protein GERALT targeted to stroma ((**A**), reprinted from [17] under Creative Commons CC―BY―NC license) and in suspension of isolated chloroplasts of N. benthamiana plants that had been transformed with NRIP1 fused to cerulean ((**B**), reprinted from [18] under CC BY 4.0 license) followed by schematic drawing of stromule in respect to chloroplast main body ((**C**), based on [19]). The CLSM image (**A**) is an overlay of the green channel, representing emission of GFP, the blue channel, representing DAPI―labelled nucleus, and the red channel, representing chlorophyll emission of chloroplasts. Note that the chlorophyll emission of stromules forming a chloroplast is masked by green color only in the overlay presentation. Scale bar―2 µm. The image (**B**) is the Z-projection of CLSM images, combining chlorophyll emission (red) and cerulean emission (white) with arrows pointing to individual stromules. Scale bar—10 µm. In the schematic representation (**C**), the hypothetical possibility of unequal participation of inner and outer chloroplast envelope in stromule formation (see discussion in the text) is indicated with question marks.

In Figure 1, I present a confocal microscopy image of stromules in leaves of *Arabidopsis thaliana* seedlings overexpressing one of stroma-targeted protein GERALT, labelled with Green Fluorescent Protein (GFP). There, stromules appear as thin green threads originating from the chloroplast. In this image, stromules are relatively long and point to the nucleus; however this is not a general feature of all known stromules [4,6,16].

**Figure 2:**
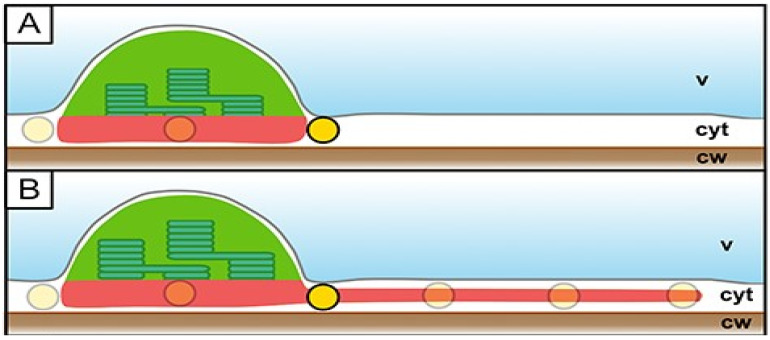
Hypothesis of stromules’ necessity for efficient plastid interaction in the quasi-2D cytoplasm of epidermal cells. (**A**) Plastids (green) in the cytoplasm (cyt) of whose cells are pressed by the vacuole (v) to the cell wall (cw). Formation of stromules (**B**) increases the area of interaction with other organelles, represented by yellow circles. Reproduced from [23] under Creative Commons Attribution License (https://creativecommons.org/licenses/by/4.0/, 30 October 2025).

In the situation presented in Figure 1, stromules are formed by chloroplasts, which is characteristic of green tissues. In general, stromules are extensions of plastids and are therefore found predominantly in photosynthetic tissues containing chloroplasts, as well as in fruits [20], containing chromoplasts. Stromules have also been observed in etiolated plants as tube-like structures connecting etioplasts [21]. However, these structures tend to vanish upon exposure to light, in contrast to normal stromules, which are induced by light. Numerous stromules formed by the chloroplasts were observed in the pavement tissue/epidermis. The cells of this tissue are thinner than mesophyll cells, and contain much lower number of chloroplasts [22]. Pavement cell chloroplasts (PCC), described in multiple plant species, are significantly smaller than mesophyll chloroplasts and have a higher stroma-to-thylakoid ratio. This feature is proposed to be the reason for the easier observation of fluorescent stromal proteins in epidermal than in mesophyll chloroplasts. PCCs exhibit a transient association with peroxisomes and mitochondria [22]; however, they are for nonethless photosynthetically active. The presence of PCCs in *Arabidopsis thaliana* has been questioned for a long time, and the observed plastids in *Arabidopsis* epidermis have been regarded as leucoplasts [22]. Sometimes it seems that PCC are the only stromules forming plastids, though this may simply reflect higher accumulation of stroma-targeted fluorescent proteins. Quite recently, Erickson et al. proposed that stromules are necessary for optimal spatial regulation in pavement cells due to their quasi-2D geometry (Figure 2), namely, very thin cytoplasm [23]. On the other hand, some Authors suggest [1] that mesophyll chloroplasts do not form stromules due to high space occupancy, and virtually no place for stromules formation. It is certainly true, that it would be much harder to find stromules in the tight spaces of mesophyll cytoplasm than in pavement cells.

The term “stromule” originates from stroma, being a designation of the chloroplast interior―specifically, the space enclosed by the inner chloroplast envelope, and located outside of thylakoids. The stroma contains the enzymatic machinery of the Calvin cycle, the stromal components of the photosynthetic electron transfer chain (such as ferredoxin and ferredoxin:NADP+ oxidoreductase), the chloroplastic transcription and translation machinery (including ribosomes), and other components responsible for functions of chloroplasts, considered as non―photosynthetic (synthesis of heme and its derivatives, isoprenoids, iron―sulphur cluster, and lipids). Within the stroma, there are also thylakoids―vesicles of photosynthetic membranes. An individual thylakoid, when isolated, is about 100 nm in diameter; however, within a chloroplast, they assemble into stacks (grana) of about 300 nm diameter and about 200 nm height. Grana are interconnected by thylakoids of the stroma, being an extended tubular version of the sacs.

Typically, stromules are visualized using fluorescent proteins (such as GFP) targeted to the chloroplast stroma via various transit peptides, adapted from known stromatargeted proteins (such as ferredoxin:NADP+ oxidoreductase, FNR, or small subunit of RuBisCo, RbcS). It is then logical that tubular extension filled with such proteins should be wrapped in outer and inner chloroplast membranes. Although this assumption seems to be broadly accepted in the literature, direct experimental evidence is missing so far. The information was provided as unpublished data, based on fluorescently labelled outer and inner membranes by Gray et al. [2]. Stronger evidence came from TEM studies; however, this was done on non―non-photosynthetic plastids. Interestingly, in our preliminary studies, labeling the outer and inner chloroplast envelope of *Nicotiana benthamiana* chloroplast, we found several stromules not containing the inner chloroplast membrane (unpublished). However, in this study, we have not yet included labeling of stroma, and the observed stromules may be an initial stage of stromule formation (sometimes called stromulation), rather than mature stromules. It is worth noting here that both visualization and detection of stromules in various situations, may depend strongly on the chosen label. Because fluorescent labelling of stromules demands genetic manipulation, there is always a possibility that the native system of the plant cell was impaired. It is especially possible with overexpression, leading sometimes to artificial protein clustering. Moreover, transient transformation, a common techniquein stromules studies, involves introducing *Agrobacterium* into a plant tissue. This is a situation that resembles a pathogen infection, and should therefore be considered when interpreting results. Finally, fluorescence-based methods have their limitations. The most important is the sensitivity of the fluorescence signal to environmental conditions, such as pH or interaction with other factors, that may, for example, change the conformation of the fluorophore. The fluorescence signal detection might be challenging due to the background of autofluorescence, particulalry when native promoter instead of stronger once is used.

The assumption that stromules are cenclosed by the inner chloroplast envelope is logical and strongly supported by stromules filling with stroma-targeted proteins. The inner envelope of the chloroplast lacks large channels, allowing the stroma content to penetrate into the intermembrane space (see further text). However, it is noteworthy that thylakoids—although present in the stroma—have never been shown in stromules. With the accepted stromules’ diameter, one may expect that thylakoids should also extend into stromules; however, chlorophyll emission hasn’t been detected in those structures so far. This fact is related to the organization of thylakoids into a counteracting net of grana and lamella. Thylakoids are kept together by a combination of electrostatic interactions, van der Waals attraction, hydration force, steric hindrance, and some specific interactions so far unlikely to release a single vesicle [24].

In this Review, I will focus on a key question, whether stromules—which are not always present in plant cells—might serve as formalized pathways to transfer a signal to the nucleus. A formalized pathway will be understood here as a conduit for any molecule, including small molecules and proteins. Although the Reader may find a more exhaustive collection of the available knowledge, describing all aspects of stromules occurrence, I would like to critically assess the problem from a biophysical perspective. Specifically, I will examine the mechanism of stromules formation (namely, may it is random if its creation involves high energetic costs and probable specialized machinery) and the possibility of transport via these routes.

## 2. How They Began

In green tissues, chloroplasts stromules are created in response to light [16,25,26], as well as to sugars, a direct metabolite of photosynthesis. The last was demonstrated, e.g., by Schattat and Klosgen [27], who investigated a sugar factor, inducing stromule formation in leaf tissue of Arabidopsis. They used a leaf epidermis from plants with stable expression of GFP targeted to the chloroplast stroma with FNR target peptide. Leaf cuts were vacuum infiltrated with sugars or inhibitors and incubated in darkness. Stromule induction was observed after 1 h, with a maximum at 2 h after treatment with sucrose and glucose. Weaker effects were detected for mannitol and fructose, whereas prolonged exposure to sorbitol had a negative impact. Optimal sucrose concentration was about 40 mM, which was later used in other studies to successfully induce stromules [28]. Sucrose-induced stromules formation was shown to depend on protein biosynthesis, both in the cytoplasm and in the chloroplast. This was proven by inhibition of the process with cyclohexamide, an inhibitor of eukaryotic protein biosynthesis, while prokaryotic inhibitors of protein biosynthesis, streptomycin and spectinomycin, modified stromule formation kinetics [27,29]. In another experiment, low-phosphate growth conditions promoted stromules formation, with involvement of strigolactones [30], a class of plant phytohormones crucial for establishing mycorrhizatype interactions between plants and fungi.

It is a general observation that there exist a strong connection between stromule formation and exposure to abiotic and biotic stress. The proof for that is a study of Gray et al., where the stromules were induced by precursors of ethylene and treatment with methyl jasmonate, while salicylic acid prevented stromules’ appearance [29]. Also, direct application of abscisic acid can induce stromules [29]. Morover, two abiotic stress-related proteins, ACCLIMATION OF PHOTOSYNTHESIS TO ENVIRONMENT1 (APE1) and ATG8-INTERACTING PROTEIN1 (ATI1), were shown to form a dimer, which was later postulated as necessary for stromules induction [31]. The connection between stromule formation and the chloroplast redox state was proven by observation of increased stromule frequency after silencing thioredoxin reductase, one of key enzymes for redox balance in the stroma [26]. Some periodicity in the frequency of stromule formation was observed in a day cycle [32]. Notably, stromules might also be formed by isolated chloroplasts, in a manner independent of stress factors [16,18,26].

Recently, there were also a series of papers that suggest that stromules primarily function in plant immune response [8]. To support that, a group of Dinesh―Kumar showed that kinesin KIS1 (KINESIN REQUIRED FOR INDUCING STROMULES 1) was necessary for stromules formation in immune response, e.g., under tobacco mosaic virus infection, via Toll/Interleukin―1 receptor―type nucleotide―binding leucine―rich repeat―immune receptor―mediated pathways of immune response [33]. KIS1 protein contains a caloponin homology domain that binds actin. Additionally, in those experiments, KIS―1―dependent stromules formation required early immune signaling components ENHANCED DISEASE SUSCEPTIBILITY 1 (EDS1) and PHYTOALEXIN DEFICIENT 4 (PAD4) [33]. The same group demonstrated stromules induction by endogenously applied hydrogen peroxide, one of the universal signals in plant immune response [9]. These experiments provided evidence for the transfer of a protein, chloroplast localized NRIP1 defense protein, through stromules from chloroplasts to the nucleus. Similarly, *Phytophtora* infection was found to induce stromules via BRASSINOSTEROID INSENSITIVE 1―ASSOCIATED KINASE 1 (BAK1) dependent signaling [34].

Interestingly, Krenz et al. [35] reported that they were unsuccessful in observing stromules in the absence of infection, which is in contradiction with our laboratory observation [17] and many other literature reports (see, e.g., [31,36]). Such discrepancies may reflect differences between stromules’ induction mechanisms, even in the same plant, but under different conditions. The crucial factor, as mentioned earlier, might be labeling, used for stromules visualization. Although typical stroma labeling is done with the use of fluorescent proteins, which are generally believed to be neutral and not interacting with chloroplast components, it cannot be excluded that, if stromules trafficking involves active transport, these proteins may not efficiently enter the stromules.

In general, formation of stromules is observed within tens of minutes [18]. It begins as a bulge on the chloroplast surface that extends further ([19], Figure 1C). Descriptions in literature suggest a scenario in which the stromule’s diameter is defined by this primary bulge size and no further broadening takes place [12,16,18,26]. What happens is the elongation of protrusion, with occasional branching [23,27,37]. It is therefore plausible that the primary bulge structure is formed with the involvement of some stabilizing protein elements, like a protein ring of roughly defined size. Some studies, however, describe stromulelike structures (especially found in fruits) that are present in tissue without exposure to any known stromule inducing factors. Pyke and Howels [20] observed stromules in tomato. They were found in epidermal cells, trichomes of stems, and petioles. Also, the pericarp cells’ chromoplasts had formed interconnecting stromules. The stromules were visualized using the RecA-GFP construct, targeting to chloroplast/chromoplast stroma. Chloroplast and stromules were analysed by confocal fluorescence microscopy; however, the resulting images were of considerably low quality (the interior of the chloroplast is solid chlorophyll fluorescence), which did not allow for to analysis of chloroplast morphology except identification of tubular extension. Stromules of trichomes displayed irregularities and swelling/beading along the main tubule.

The stromules creation mechanisms are, in principle, similar to the formation of a dynamic tubular network of endoplasmic reticulum (ER), although these tubules are generally thinner (30–60 nm in diameter). ER tubules are shaped due to to the presence of reticulons and REEPs/Yop1p, a class of transmembrane proteins that induce high membrane curvature [38]. The induction of membrane shape is due to the hairpin conformation of proteins, which occupies more space in the outer membrane leaflet than in the inner leaflet. In plants, reticulon proteins were shown to oligomerize [39]. A similar high-curvature step is present during nuclear pore formation [40]. Within an ER tubule, the luminal space is stabilized by dimerisation of proteins (e.g., Climp63), forming clip―like structures that maintain spacing between tubule walls [41].

Stromules’ association with microtubules, as well as reorganization of stromules in response to a dynamic change in the microtubule network, were shown [21]. Kumar et al. [42] observed that in *N. tabacum* the stromules interact with microtubules, with stromules branching accompanying microtubules crossing. Knockout of CHUP1 (CHLOROPLAST UNUSUAL POSITIONING1), a protein of the outer chloroplast envelope, resulted in constitutive stromule formation [9]. CHUP1 is a protein that is necessary for chloroplast anchorage to the plasma membrane and adaptational movement [43]. Nedo et al. investigated the mechanism connecting CHUP1 and stromule formation. They showed that without CHUP1 in the chloroplast membrane, stromules extend faster; however moment of induction is CHUP1independent. In light of that, anchoring of chloroplast membranes to the cytoskeleton, via CHUP1, may work as a restriction mode, organizing membrane extension in the desired direction [44].

It was proposed that stromule formation might be due to hydrostatic pressure; however, just an increase of osmotic pressure inside chloroplasts would result in an increase of chloroplast volume and breaking of the chloroplast envelope, rather than the formation of a small nodule. Such swelling might be achieved by putting isolated chloroplasts in a hypoosmotic medium or in a solution of ammonia [45,46,47]. In none of those conditions were stromule formation observed.

Small tubules are also formed by mitochondria, and they are called matrixules [13]. There were also shown peroxisome extensions, called peroxisules [13]. J. Mathur proposed to use a group name of “organelles extension” and arguments for ER―driven dynamics of all these structures [12]. This again raises the question of the involvement of both chloroplast envelopes in the formation of stromules. While mitochondria are double membrane organelles, ER and peroxisomes are not. The outer envelope of both chloroplasts and mitochondria is more similar in compositing to the ER than to the inner membrane. There are mitochondria associated ER membranes [48]. Recently, some luminal vesicularisation was observed for peroxisomes, with the possibility of compartmentalization [49]. Such behavior demonstrates high flexibility of membranes, but again shows that spontaneous vesiculation results in nontubular shapes. The spherical vesicles are a result of thermodynamically favoured shape adapted by lipid bilayers in a water environment, and their change to a tubular form needs energy and dedicated protein components. Morover, tubular extension of organelles, especially stromules, is formed within ten minutes, and their formation cannot rely on intensive de novo protein synthesis.

Mitochondria’s situation is different from chloroplasts, as they are highly dynamic [50], with their fusion-fission cycles, resulting in division and connection of membranes. It is reasonable to postulate that mitochondria interconnecting tubules may be a remnant of the division process, but if such a tubule is directed to the nucleus or chloroplast cannot be that remnant, but must be formed in response to some stimulus. In contrast, division of chloroplasts in mature tissue is very rare, and cannot explain the induction of stromules by light.

## 3. The Stromules and Chloroplast Envelope Membranes

When discussing the nature of tubular extensions among different plastid types, one must first consider their envelope membrane properties. Are elasticity and composition of the envelope comparable across all plastids? The composition and biophysical properties of the etioplast envelope remain poorly characterised. It is believed that they are converted later into the chloroplast envelope, with their specific function in translocation and biosynthesis; however, more detailed studies are missing.

With no doubt, stromule formation requires an excess of membrane material [37]. This implies that membrane components should either be newly synthesized or recruited from preexisting storage. The membrane proteins must be primarily synthesized, mostly in the cytosol, which was evidenced by the blockage of stromules formation with inhibitors of protein biosynthesis [27,29]. The second essential membrane component —lipids —may, to some extent, be recruited from plastoglobules, lipid-rich structures connected to thylakoids. One, although not only, function of plastoglubules is being a lipid reservoirs for membrane remodeling [51,52]. Plastoglobules were shown to be subject to change under exposure to various stressors [53,54]; therefore, in the conditions of stromules induction. However, an exchange of material between plastoglobules and stromules has not been investigated so far.

Chloroplast membranes are prone to forming high curvature elements. This is particularly evident in thylakoid membranes, which are believed to keep their curvature due to lipid composition and the presence of shaping proteins. The most critical lipid in this content is monogalactosylglycerol (MGDG); this lipid is characterized by a relatively small hydrophilic head group, therefore destabilizing a bilayer. MGDG is present in high content in thylakoids (about 57% of total lipids); however, inner and outer envelopes also contain it (55% and 27%, respectively) [55]. It is accepted that the composition of the outer envelope is somehow a result of this membrane interaction, both with chloroplastic membranes and the ER. Indeed, one of the MGDG synthases is associated with outer chloroplast membranes [55]. Importantly, chloroplasts lack large, nuclear pore―like opening. Consequently, if there is no easy―to―pass pore, then transfer of protein factors, is there is some—must rely on specific transporters, or membrane junction/pore formation in contact with the targeted organelle. The outer chloroplast membrane must be in direct contact with the ER, as both share lipid synthesis pathways.

Since stromules are defined as extensions of various plastid types, it is worth reminding plastid types and the specificity of their envelope membranes. In germ, hidden in the seed, there are proplastids. Development of further types of plastids depends on the fate of the tissue. After exposure to light, proplastids convert into chloroplasts. However, if the development of the seedling is carried out in darkness, there are etioplasts formed. Etioplasts are characterized by the presence of a huge structure, called a prolamellar body, that is a type of paracrystalline structure composed of thylakoid lipids and an enzyme, protochloprophyllide oxidoreductase. Upon illumination, within a few hours, etioplasts are converted into chloroplasts [56]. Therefore, the envelope of plastids is of interest when discussing the stromule creation mechanism. The outer membrane is believed to be permeable to molecules up to 10 kDa, as an unspecific molecular sieve [57]. It also participates in noncanonical protein transfer via channel known as OEP16, OEP21, OEP24, and OEP37 [57]. The restriction zone of those channels is believed to be not higher than 3 nm.

In addition, chloroplasts possess very specific translocon (TIC―TOC) system, responsible for protein import into chloroplasts. This system recognizes chloroplast targeting peptides and drags the protein to the chloroplast stroma. The translocons mostly span through both outer and inner membranes, which was recently shown for *Chlamydomonas* membranes, using cryo―EM; however, alternative pathways may allow translocation into the intermembrane space only [58]. This raises the question of whether stromule formation should sustain this supercomplex stability. The inner chloroplast membrane is much more selective for transported molecules. There is a large set of α―α-helical membrane transporters.

As mentioned earlier, the potential release of proteins from stromules remains controversial, as the possibility of protein export from chloroplasts is not clear. One of the putative export routes involves the cytosolic degradation of ubiquitinylated chloroplast proteins; however, in chloroplasts, there is its own degradation system, CHLORAD [59]. Some chloroplast chloroplast―localized proteins (like GUN1) regulate nuclear gene expression, though there is no direct evidence forGUN1 export [60].

Chloroplasts, in specialized tissues, differentiate into chromoplasts, amyloplasts, leucoplasts, elaioplasts, or proteinoplasts [61]. Most of them were shown to form stromules, suggesting that the mechanism might be of inherent membrane characteristics. All mentioned types of plastids might arise directly from proplastids. In general, there is a possibility of plastid interconversion and reverse conversion [61]. During plant senescence, chloroplasts are transformed into gerontoplasts [62]. Conversion of plastid involves a substantial reorganization of the internal membrane system (e.g., thylakoids of chloroplasts lose photosynthetic function and become filled with carotenoids, when converting into chromoplasts), and modification of envelope composition. In particular, the TIC―TOC translocon is restructured to recognize different targeting peptides [63]. Mature chromoplastasts lose their chloroplast ellipsoidal shape and adopt various morphologies: globular, crystalline, tubular, and membranous [63]. Waters et al. [1] analyzed stromules morphology during the ripening of tomato fruits. The authors found that distinct chloroplasts are more prone to form stromules. The first two chloroplast populations were noticed: outer cells are rich in small, numerous chloroplasts, while the interior is populated with large, starchfilled chloroplasts. The same differences are translated into chromoplasts in ripe tissue. Longer and more often stromules were observed in internal tissue, as well as in hypocotyl tissues with fewer chloroplasts per cell. Inhibition of chloroplast differentiation by fruit darkening or by *rin* mutation resulted in increased stromule frequency [1].

Storage plastids maturation starts at the step of leucoplast, arising either directly from proplastids, or via conversion of chloroplast or chromoplast. In general, leucoplasts are non―non-photosynthetic, colorless organelles. The leucoplast envelope contains specific transporters and translocons, and is still the place for fatty acid synthesis [64]. The translocons, however, are capable to recognize different transit peptides [65]. The leucoplasts may further differentiate into amyloplasts, elaioplasts, or proteinoplasts (aleuroplasts).

Gerontoplasts are believed to be a stage of controlled chloroplast senescence, and they possess a specific set of proteins [66]. There are no dedicated studies of the gerontoplast envelope.

Amyloplasts are accumulating starch granules for spare materials in the seed endosperm. Their envelope contains specific proteins, such as SUBSTANDARD STARCH GRAIN6, which directly regulate the accumulation and growth of starch granules [67]. The reverse conversion of amyloplast to chloroplasts is well known, and characterized by degradation of starch and *de novo* synthesis of thylakoid membrane; however also lacks details about the fate of the outer envelope [68].

Another storage-like plastid is the elaioplast, responsible for lipid reserve [69]. These plastids are especially rich in plastoglobules—the small lipoprotein globular structures, filled with lipid mixture, with surface stabilized by specific proteins.

Chloroplast envelope membranes play a central role in acclimation and stress response [70]. Membrane remodeling involves both changes in lipid composition and protein pattern [71]. Stress response can occur from a few minutes to several hours. The first stage involves adjustement of membrane lipid composition, with plastoglobules being a storage site for exchange lipid molecules, as well as protein phosphorylation [72]. The longer response, including for example alteration of membrane permeability or transporter pattern, needs hours to days [73]. Remodeling of the chloroplast envelope is connected to GUN1 and SAL1―PAP signaling pathways. There is also a pathway involving proteolytic degradation of envelope bound plant homeodomain transcription factor and activation by those fragments, transcription of *Abi* genes in the nucleus. However, since this factor is an envelope membrane associated protein [74], its release from the chloroplast may not require export across a membrane.

Interestingly, abiotic stress may induce chloroplast vesiculation. In such a case, the clathrin-dependent endocytosis mechanism leads to the formation of vesicles of the inner chloroplast envelope [75]. Similarly, during senescence, vacuoles independent of autophagy pathways were described [75].

Stromule formation is highly sensitive to temperature, which may reflect the influence of membrane fluidity and remodeling. In the experiments of Holzinger et al. low temperatures (4–15 °C), almost no extensions from the chloroplast were found, while elevated temperatures (35–45 °C) promoted protrusion of the chloroplast envelope. Closer inspection of images recorded at higher temperatures reveals that the membrane is disturbed in comparison to control plants, growing at 25 °C; the surface of the chloroplast seems to contain a lot of bulges, with a membrane greatly extended and occupying much more space [36].

## 4. Are Tubular Pathways Necessary?

In eukaryotic cells, the main center of decision-making is the nucleus. There, the fates of other cellular compartments are decided simply by regulation of gene expression, resulting in varied expression of proteins. The number of proteins may be later regulated by mRNA processing, on a transcriptional level or by post-translational regulation of protein stability, but without mRNA synthesis, there is no field of action. Nuclear anterograde signaling may also include factors regulating the expression of genes, coded by chloroplastic nucleoids. Here belong PIFs (phytochrome interacting transcription factors) [76]. However, organelles, especially chloroplasts and mitochondria, are highly complex systems that are regulated by multiple feedback loops. Therefore, there needs to be a signaling pathway that informs the nucleus of the actual physiological state of a given organelle. The process, known as retrograde signaling, has been studied for over 30 years, but its mechanism is still not fully understood [77]. Involvement of GUN (genomes uncoupled) proteins, COP (constitutive morphogenic) proteins, and other factors associated with phytochrome/cryptochrome pathways has been confirmed in retrograde signaling. There are also postulated non-protein messangers, such as tetrapyrrole (e.g.,heme or chlorophyll precursor), reactive oxygen species, and chloroplast metabolites. Retrograde signaling pathways differ between dark and light conditions, as well as under various stressors [78]. The transfer of messengers may occur via diffusion through the cytoplasm, and also via special connections between chloroplasts and the nucleus, namely stromules. In a lot of observations, multiple stromules point to nucleus, strongly supporting the claim that this is one of the pathways of retrograde signaling. In a detailed study [37] on *Arabidopsis* leaf epidermis, about 90% of stromules were facing the nucleus, and the formation of stromules occurred mostly in the 8 µm zone around the nucleus. These observations led Erickson et al. [37] to hypothesize that stromules are a kind of thread that keeps a certain number of chloroplasts in proximity to the nucleus. This behavior is known as perinuclear chloroplast clustering and has been described under pathogen attacks, in senescence, and in various stresses, which proceed with reactive oxygen species formation [79]. That seems strongly unlikely when the mechanism of stromule formation is considered―there is no contact between the chloroplast membrane and the nucleus necessary to observe the protrusion of the chloroplast membrane. Still, these findings suggestthe presence of factors polarizing chloroplast bulging. Together with a zone for a higher frequency of stromules, one may think of a gradient of a substance, released from the nucleus, that is necessary for stromulation. This idea aligns with the observation of the stromules formation process in suspension of isolated chloroplasts, which needed a presence of a not―yet―identified cytosol component, bigger than 100 kDa [18]. Consistently, Kunjumon et al. [19] investigated perinuclear chloroplast clustering under stress conditions and normal growth, finding that stromules are not necessary to keep chloroplasts near to nucleus. Instead, the clustering appears to be driven by changes in the endoplasmic reticulum. They also showed that stromules’ orientation towards the nucleus requires some light-dependent reactions, since sugar suplying to dark―adapted plants did not restore directionality of stromules [19]. Early stromules studies proposed that stromules anchor plastids to the plasma membrane [80]. Currently, it seems unlikely, and those observations might be interpreted as a result of strong stromulation tendency, resulting in multiple stromules, at the early stage of seedling development.

Considering all the available data, it is reasonable to propose that stromules should function as a fast communication track for delivery from the chloroplast to the nucleus [7]. Such a transfer was proposed to function for hydrogen peroxide [6]. Recently, the transfer via stromules was shown for photomorphogenesis regulators, NUCLEAR CONTROL OF PEP ACTIVITY [81]. The formalized tubular pathway will be faster than diffusion through cytoplasm.

The main question, however, remains:what factors are transferred by stromules—are they only small molecules, like hydrogen peroxide, or proteins? Hanson and Sattarzadeh [82] adressed this with mEOS targeted to chloroplasts, taking advantage of the photoconvertability of this protein, to demonstrate protein transport between two chloroplasts via stromules, by a distance as long as 40 µm. The results from the Schwille group [83], obtained with direct probing of molecular velocity by fluorescence correlation spectroscopy, further indicate that GFP may diffuse within stromules, sometimes in small packets of several molecules. Also, some suggestions of active transport through a stromule tube have been shown [83]. Therefore, it is safe to postulate that stromules are indeed a formalized pathway, at least between the chloroplast and the nucleus, formed within a plant cell under specific physiological conditions that demand a fast track for communication. Such a situation is a pathogen attack, as well as abiotic stress. There is also a physiological state of illumination, which may result in light stress, that needs alteration in the nuclear gene expression profile. In all such cases, it is possible to deliver a signal (like hydrogen peroxide, polyunsaturated fatty acids, and other signaling molecules) through the cytoplasm, relying on diffusion. However, diffusion is, in principle, non―directional, which requires a relatively high concentration of messenger to reach its destination. Additionally, the rate of diffusion is restricted by properties of cytoplasm and molecular crowding and the presence of scavengers. In both cases, dedicated stromules may facilitate faster and more effective communication. With this argumentation, there is still a question of messenger release. There is no doubt that small molecules may pass the chloroplast envelope, while the release of protein would demand not yet recognized transporters of pores. The same problem, however, will be noted for any proteintype messenger release from chloroplasts; therefore, this point cannot be provided as the argument against stromules’ involvement in signaling.

## 5. Conclusions

**Figure 3:**
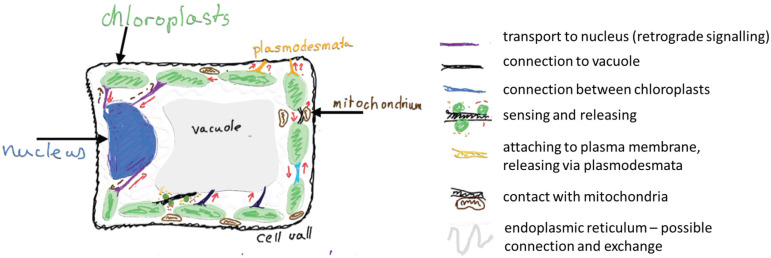
Schematic summary of the proposed functions of stromules in a photosynthetic cell. Red arrows suggest the direction of information exchange, brown arrow with question mark indicate additional putative routes. Own creation, inspired by [6,8].

Stromules are tubular extensions of chloroplast that may serve as a fast track for information exchange within a plant cell (Figure 3). In this short review, I have presented arguments, primarily from a biophysical perspective, both for and against the idea that stromules function as a formalized communication pathway. Although the Reader may see that my interpretation favors hypothesis favors the intentional appearance of stromules, the stromules’ actual function remains a matter of debate. On one hand, stromules formation is typically observed under specific physiological conditions (intensive photosynthesis, pathogens attack) and their formation demands allocation of resources (lipids and proteins for membrane extension). On the other hand, at least in some studies, stromules were found to resemble ER behavior and not be able to exchange their content with the neighbouring organelles. It is possible that all these contradictory data are the result of different types of stromules, and still missing a detailed description of stromules’ content.

## Data Availability

Not applicable.

## References

[1] Waters M.T., Fray R.G., Pyke K.A. (2004). Stromule formation is dependent upon plastid size, plastid differentiation status and the density of plastids within the cell. Plant J..

[2] Gray J., Sullivan J., Hibberd J., Hansen M. (2001). Stromules: Mobile protrusions and interconnections between plastids. Plant Biol..

[3] Hanson M.R., Hines K.M. (2018). Stromules: Probing formation and function. Plant Physiol..

[4] Natesan S.K.A., Sullivan J.A., Gray J.C. (2005). Stromules: A characteristic cell-specific feature of plastid morphology. J. Exp. Bot..

[5] Kwok E., Hanson M. (2004). Stromules and the dynamic nature of plastid morphology. J. Microsc..

[6] Hanson M.R., Conklin P.L. (2020). Stromules, functional extensions of plastids within the plant cell. Curr. Opin. Plant Biol..

[7] Mullineaux P.M., Exposito-Rodriguez M., Laissue P.P., Smirnoff N., Park E. (2020). Spatial chloroplast-to-nucleus signalling involving plastid–nuclear complexes and stromules. Philos. Trans. R. Soc. B.

[8] Jung S., Woo J., Park E. (2024). Talk to your neighbors in an emergency: Stromule-mediated chloroplast-nucleus communication in plant immunity. Curr. Opin. Plant Biol..

[9] Caplan J.L., Kumar A.S., Park E., Padmanabhan M.S., Hoban K., Modla S., Czymmek K., Dinesh-Kumar S.P. (2015). Chloroplast stromules function during innate immunity. Dev. Cell.

[10] Huang J., Taylor J.P., Chen J.-G., Uhrig J.F., Schnell D.J., Nakagawa T., Korth K.L., Jones A.M. (2006). The plastid protein THYLAKOID FORMATION1 and the plasma membrane G-protein GPA1 interact in a novel sugar-signaling mechanism in Arabidopsis. Plant Cell.

[11] Michaud M., Jouhet J. (2019). Lipid trafficking at membrane contact sites during plant development and stress response. Front. Plant Sci..

[12] Mathur J., Kroeker O.F., Lobbezoo M., Mathur N. (2022). The ER is a common mediator for the behavior and interactions of other organelles. Front. Plant Sci..

[13] Mathur J. (2021). Organelle extensions in plant cells. Plant Physiol..

[14] Schattat M.H., Barton K.A., Mathur J. (2015). The myth of interconnected plastids and related phenomena. Protoplasma.

[15] Baillie A.L., Falz A.-L., Müller-Schüssele S.J., Sparkes I. (2020). It started with a kiss: Monitoring organelle interactions and identifying membrane contact site components in plants. Front. Plant Sci..

[16] Brunkard J.O., Runkel A.M., Zambryski P. (2016). Visualizing stromule frequency with fluorescence microscopy. J. Vis. Exp..

[17] Banas A., Grzyb J., Zglobicki P., Pacak A., Mysliwa-Kurdziel B., Leja K., Kozieradzka-Kiszkurno M., Klodawska K., Konieczny R., Pilarska M. (2025). GERALT, A Cryptochrome/Photolyase Family Protein, Is Essential for Young Chloroplast Development and Function with Its Importance Decreasing in Older Plants. Plant Cell Physiol..

[18] Ho J., Theg S.M. (2016). The formation of stromules In Vitro from chloroplasts isolated from *Nicotiana benthamiana*. PLoS ONE.

[19] Kunjumon T.K., Ghosh P.P., Currie L.M., Mathur J. (2024). Proximity driven plastid–nucleus relationships are facilitated by tandem plastid–ER dynamics. J. Exp. Bot..

[20] Pyke K.A., Howells C.A. (2002). Plastid and stromule morphogenesis in tomato. Ann. Bot..

[21] Kwok E.Y., Hanson M.R. (2004). In vivo analysis of interactions between GFP-labeled microfilaments and plastid stromules. BMC Plant Biol..

[22] Barton K.A., Schattat M.H., Jakob T., Hause G., Wilhelm C., Mckenna J.F., Máthé C., Runions J., Van Damme D., Mathur J. (2016). Epidermal pavement cells of Arabidopsis have chloroplasts. Am. Soc. Plant Biol..

[23] Erickson J.L., Prautsch J., Reynvoet F., Niemeyer F., Hause G., Johnston I.G., Schattat M.H. (2024). Stromule geometry allows optimal spatial regulation of organelle interactions in the quasi-2D cytoplasm. Plant Cell Physiol..

[24] Chow W.S., Kim E.-H., Horton P., Anderson J.M. (2005). Granal stacking of thylakoid membranes in higher plant chloroplasts: The physicochemical forces at work and the functional consequences that ensue. Photochem. Photobiol. Sci..

[25] Itoh R.D. (2023). Tubular extensions of plant organelles and their implications on retrograde signaling. J. Biol. Res.-Boll. Soc. Ital. Biol. Sper..

[26] Brunkard J.O., Runkel A.M., Zambryski P.C. (2015). Chloroplasts extend stromules independently and in response to internal redox signals. Proc. Natl. Acad. Sci. USA.

[27] Schattat M.H., Klösgen R.B. (2011). Induction of stromule formation by extracellular sucrose and glucose in epidermal leaf tissue of *Arabidopsis thaliana*. BMC Plant Biol..

[28] Delfosse K., Wozny M.R., Barton K.A., Mathur N., Griffiths N., Mathur J. (2018). Plastid envelope-localized proteins exhibit a stochastic spatiotemporal relationship to stromules. Front. Plant Sci..

[29] Gray J.C., Hansen M.R., Shaw D.J., Graham K., Dale R., Smallman P., Natesan S.K., Newell C.A. (2012). Plastid stromules are induced by stress treatments acting through abscisic acid. Plant J..

[30] Vismans G., van der Meer T., Langevoort O., Schreuder M., Bouwmeester H., Peisker H., Dörman P., Ketelaar T., van der Krol A. (2016). Low-phosphate induction of plastidal stromules is dependent on strigolactones but not on the canonical strigolactone signaling component MAX2. Plant Physiol..

[31] Ge Z., Jing Y., Zhu J., Yang L.-E., Lu S., Deng Y. (2025). APE1 localizes to chloroplast stromules and interacts with ATI1 in Arabidopsis. Plant Signal. Behav..

[32] Shimahara Y., Kutsuna N., Hasezawa S., Kojo K.H. (2019). Quantitative evaluation of stromule frequency at hourly intervals in Arabidopsis stomatal guard cell chloroplasts. Cytologia.

[33] Meier N.D., Seward K., Caplan J.L., Dinesh-Kumar S.P. (2023). Calponin homology domain containing kinesin, KIS1, regulates chloroplast stromule formation and immunity. Sci. Adv..

[34] Savage Z., Duggan C., Toufexi A., Pandey P., Liang Y., Segretin M.E., Yuen L.H., Gaboriau D.C., Leary A.Y., Tumtas Y. (2021). Chloroplasts alter their morphology and accumulate at the pathogen interface during infection by *Phytophthora infestans*. Plant J..

[35] Krenz B., Guo T.W., Kleinow T. (2014). Stromuling when stressed!. Acta Soc. Bot. Pol..

[36] Holzinger A., Buchner O., Lütz C., Hanson M. (2007). Temperature-sensitive formation of chloroplast protrusions and stromules in mesophyll cells of *Arabidopsis thaliana*. Protoplasma.

[37] Erickson J.L., Schattat M.H. (2018). Shaping plastid stromules—Principles of in vitro membrane tubulation applied in planta. Curr. Opin. Plant Biol..

[38] Wang X., Li S., Wang H., Shui W., Hu J. (2017). Quantitative proteomics reveal proteins enriched in tubular endoplasmic reticulum of *Saccharomyces cerevisiae*. eLife.

[39] Breeze E., Dzimitrowicz N., Kriechbaumer V., Brooks R., Botchway S.W., Brady J.P., Hawes C., Dixon A.M., Schnell J.R., Fricker M.D. (2016). A C-terminal amphipathic helix is necessary for the in vivo tubule-shaping function of a plant reticulon. Proc. Natl. Acad. Sci. USA.

[40] Dultz E., Wojtynek M., Medalia O., Onischenko E. (2022). The nuclear pore complex: Birth, life, and death of a cellular behemoth. Cells.

[41] Xu L., Xiang Y., Hu J. (2023). Molecular basis of Climp63-mediated ER lumen spacing. J. Cell Sci..

[42] Kumar A.S., Park E., Nedo A., Alqarni A., Ren L., Hoban K., Modla S., McDonald J.H., Kambhamettu C., Dinesh-Kumar S.P. (2018). Stromule extension along microtubules coordinated with actin-mediated anchoring guides perinuclear chloroplast movement during innate immunity. eLife.

[43] Oikawa K., Yamasato A., Kong S.-G., Kasahara M., Nakai M., Takahashi F., Ogura Y., Kagawa T., Wada M. (2008). Chloroplast outer envelope protein CHUP1 is essential for chloroplast anchorage to the plasma membrane and chloroplast movement. Plant Physiol..

[44] Nedo A.O., Liang H., Sriram J., Razzak M.A., Lee J.Y., Kambhamettu C., Dinesh-Kumar S.P., Caplan J.L. (2024). CHUP1 restricts chloroplast movement and effector-triggered immunity in epidermal cells. New Phytol..

[45] Kaiser W.M., Stepper W., Urbach W. (1981). Photosynthesis of isolated chloroplasts and protoplasts under osmotic stress: Reversible swelling of chloroplasts by hypotonic treatment and its effect on photosynthesis. Planta.

[46] Block M.A., Albrieux C., Maréchal E. (2024). Purification of Chloroplast Envelope, Thylakoids, and Stroma from Angiosperm Leaves. Plastids: Methods and Protocols.

[47] Schuldiner S., Avron M. (1971). Anion permeability of chloroplasts. Eur. J. Biochem..

[48] Mueller S.J., Reski R. (2015). Mitochondrial dynamics and the ER: The plant perspective. Front. Cell Dev. Biol..

[49] Wright Z.J., Bartel B. (2020). Peroxisomes form intralumenal vesicles with roles in fatty acid catabolism and protein compartmentalization in Arabidopsis. Nat. Commun..

[50] Chustecki J.M., Gibbs D.J., Bassel G.W., Johnston I.G. (2021). Network analysis of Arabidopsis mitochondrial dynamics reveals a resolved tradeoff between physical distribution and social connectivity. Cell Syst..

[51] Austin J.R., Frost E., Vidi P.-A., Kessler F., Staehelin L.A. (2006). Plastoglobules are lipoprotein subcompartments of the chloroplast that are permanently coupled to thylakoid membranes and contain biosynthetic enzymes. Plant Cell.

[52] Rottet S., Besagni C., Kessler F. (2015). The role of plastoglobules in thylakoid lipid remodeling during plant development. Biochim. Biophys. Acta (BBA)-Bioenerg..

[53] Ying S. (2025). Get the Ball Rolling: Update and Perspective on the Role of Chloroplast Plastoglobule-associated Protein under Abiotic Stress. J. Exp. Bot..

[54] Coulon D., Nacir H., Bahammou D., Jouhet J., Bessoule J.-J., Fouillen L., Bréhélin C. (2024). Roles of plastoglobules and lipid droplets in leaf neutral lipid accumulation during senescence and nitrogen deprivation. J. Exp. Bot..

[55] Block M.A., Douce R., Joyard J., Rolland N. (2007). Chloroplast envelope membranes: A dynamic interface between plastids and the cytosol. Photosynth. Res..

[56] Banaś A.K., Leja K., Zgłobicki P., Jedynak P., Kowalska E., Strzałka W., Grzyb J., Myśliwa-Kurdziel B. (2024). De-etiolation is almost color blind: The study of photosynthesis awakening under blue and red light. Plant Cell Physiol..

[57] Breuers F., Bräutigam A., Weber A.P. (2011). The plastid outer envelope–a highly dynamic interface between plastid and cytoplasm. Front. Plant Sci..

[58] Jin Z., Wan L., Zhang Y., Li X., Cao Y., Liu H., Fan S., Cao D., Wang Z., Li X. (2022). Structure of a TOC-TIC supercomplex spanning two chloroplast envelope membranes. Cell.

[59] van Wijk K.J., Adam Z. (2024). Does the polyubiquitination pathway operate inside intact chloroplasts to remove proteins?. Plant Cell.

[60] Colombo M., Tadini L., Peracchio C., Ferrari R., Pesaresi P. (2016). GUN1, a jack-of-all-trades in chloroplast protein homeostasis and signaling. Front. Plant Sci..

[61] Choi H., Yi T., Ha S.-H. (2021). Diversity of plastid types and their interconversions. Front. Plant Sci..

[62] Knudsen C., Gallage N.J., Hansen C.C., Møller B.L., Laursen T. (2018). Dynamic metabolic solutions to the sessile life style of plants. Nat. Prod. Rep..

[63] Zeng Y., Du J., Wang L., Pan Z., Xu Q., Xiao S., Deng X. (2015). A comprehensive analysis of chromoplast differentiation reveals complex protein changes associated with plastoglobule biogenesis and remodeling of protein systems in sweet orange flesh. Plant Physiol..

[64] Eastmond P.J., Dennis D.T., Rawsthorne S. (1997). Evidence that a malate/inorganic phosphate exchange translocator imports carbon across the leucoplast envelope for fatty acid synthesis in developing castor seed endosperm. Plant Physiol..

[65] Chu C.-C., Li H.-m. (2025). Identification of transit peptides that boost plastid protein import in different tissues and plant species. Nat. Plants.

[66] Shah M., Soares E.L., Lima M.L., Pinheiro C.B., Soares A.A., Domont G.B., Nogueira F.C., Campos F.A. (2016). Deep proteome analysis of gerontoplasts from the inner integument of developing seeds of Jatropha curcas. J. Proteom..

[67] Matsushima R., Maekawa M., Kusano M., Tomita K., Kondo H., Nishimura H., Crofts N., Fujita N., Sakamoto W. (2016). Amyloplast membrane protein SUBSTANDARD STARCH GRAIN6 controls starch grain size in rice endosperm. Plant Physiol..

[68] Altamura M.M., Piacentini D., Della Rovere F., Fattorini L., Valletta A., Falasca G. (2024). Transition dynamics in plastid interconversion in land plants. Plant Biosyst.-Int. J. Deal. All Asp. Plant Biol..

[69] Zhu M., Lin J., Ye J., Wang R., Yang C., Gong J., Liu Y., Deng C., Liu P., Chen C. (2018). A comprehensive proteomic analysis of elaioplasts from citrus fruits reveals insights into elaioplast biogenesis and function. Hortic. Res..

[70] John A., Keller I., Ebel K.W., Neuhaus H.E. (2025). Two critical membranes: How does the chloroplast envelope affect plant acclimation properties?. J. Exp. Bot..

[71] Mamaeva A., Taliansky M., Filippova A., Love A.J., Golub N., Fesenko I. (2020). The role of chloroplast protein remodeling in stress responses and shaping of the plant peptidome. New Phytol..

[72] Jarvis P., López-Juez E. (2013). Biogenesis and homeostasis of chloroplasts and other plastids. Nat. Rev. Mol. Cell Biol..

[73] Tachibana R., Abe S., Marugami M., Yamagami A., Akema R., Ohashi T., Nishida K., Nosaki S., Miyakawa T., Tanokura M. (2024). BPG4 regulates chloroplast development and homeostasis by suppressing GLK transcription factors and involving light and brassinosteroid signaling. Nat. Commun..

[74] Sun X., Feng P., Xu X., Guo H., Ma J., Chi W., Lin R., Lu C., Zhang L. (2011). A chloroplast envelope-bound PHD transcription factor mediates chloroplast signals to the nucleus. Nat. Commun..

[75] Pan T., Liu Y., Hu X., Li P., Lin C., Tang Y., Tang W., Liu Y., Guo L., Kim C. (2023). Stress-induced endocytosis from chloroplast inner envelope membrane is mediated by CHLOROPLAST VESICULATION but inhibited by GAPC. Cell Rep..

[76] Hwang Y., Han S., Yoo C.Y., Hong L., You C., Le B.H., Shi H., Zhong S., Hoecker U., Chen X. (2022). Anterograde signaling controls plastid transcription via sigma factors separately from nuclear photosynthesis genes. Nat. Commun..

[77] Richter A.S., Nägele T., Grimm B., Kaufmann K., Schroda M., Leister D., Kleine T. (2023). Retrograde signaling in plants: A critical review focusing on the GUN pathway and beyond. Plant Commun..

[78] Hernández-Verdeja T., Strand Å. (2018). Retrograde signals navigate the path to chloroplast development. Plant Physiol..

[79] Ding X., Jimenez-Gongora T., Krenz B., Lozano-Duran R. (2019). Chloroplast clustering around the nucleus is a general response to pathogen perception in *Nicotiana benthamiana*. Mol. Plant Pathol..

[80] Kwok E.Y., Hanson M.R. (2004). Plastids and stromules interact with the nucleus and cell membrane in vascular plants. Plant Cell Rep..

[81] Lee J.-H., Doan T.M., Senthilkumar S., Yoo C.Y. (2024). Mechanism of nucleus-chloroplast communication by alternative promoter usage and stromules to establish photomorphogenesis in Arabidopsis. bioRxiv.

[82] Hanson M.R., Sattarzadeh A. (2013). Trafficking of proteins through plastid stromules. Plant Cell.

[83] Köhler R.H., Schwille P., Webb W.W., Hanson M.R. (2000). Active protein transport through plastid tubules: Velocity quantified by fluorescence correlation spectroscopy. J. Cell Sci..

